# Transport of Amino Acids in Soy Sauce Desalination Process by Electrodialysis

**DOI:** 10.3390/membranes11060408

**Published:** 2021-05-29

**Authors:** Man Wang, Shaoping Kuang, Xitong Wang, Daihao Kang, Debin Mao, Guanlan Qian, Xiaodan Cai, Ming Tan, Fei Liu, Yang Zhang

**Affiliations:** 1 College of Environment and Safety Engineering, Qingdao University of Science and Technology, 53 Zhengzhou Road, Qingdao 266042, China; 17865305301@163.com (M.W.); wangxitongg@163.com (X.W.); kangdaihao@126.com (D.K.); fliu@qust.edu.cn (F.L.); zhangyang@qust.edu.cn (Y.Z.); 2 Flemish Institute for Technological Research (VITO), Boeretang 200, BE-2400 Mol, Belgium; debin.mao@vito.be; 3 Qingdao Dengta Flavoring and Food Co., Ltd., 65 Huangtai Road, Qingdao 266012, China; qianguanlan@dengtatwp.com (G.Q.); caixiaodan@dengtatwp.com (X.C.)

**Keywords:** soy sauce, amino acids, electrodialysis, desalination, adsorption

## Abstract

Soy sauce is a common condiment that has a unique flavor, one that is derived from its rich amino acids and salts. It is known that excessive intake of high-sodium food will affect human health, causing a series of diseases such as hypertension and kidney disease. Therefore, removing sodium from the soy sauce and retaining the amino acids is desirable. In this study, electrodialysis (ED) was employed for the desalination of soy sauce using commercial ion exchange membranes (IEMs). The influence of the current density and initial pH on the desalination degree of the soy sauce was explored. Results showed that the optimal desalination condition for ED was reached at a current density of 5 mA/cm^2^ and pH of 5, with the desalination degree of 64% and the amino acid loss rate of 29.8%. Moreover, it was found that the loss rate of amino acids was related to the initial concentration and molecular structure. In addition, the amino acid adsorption by IEMs was explored. Results implied that the molecular weight and structure affect amino acid adsorption. This study illustrated that the ED process can successfully reduce the salt content of the soy sauce and retain most of the amino acids without compromising the original flavor.

## 1. Introduction

Soy sauce is a traditional condiment that is used in many Asian countries. It can bring an appetizing taste to a certain food [1]. At present, the annual production of soy sauce in the world is about 8 million tons. China is a major producer and consumer of soy sauce, with an annual export of 5 million tons [2]. Up to 18 amino acids have been found in soy sauce. These amino acids make soy sauce a healthy [3] and unique condiment. Amino acids such as valine (Val), lysine (Lys), isoleucine (Ile), leucine (Leu), phenylalanine (Phe), tryptophan (Try), and threonine (Thr) are essential for the human body. Amino acids also represent unique flavors, in which glutamic (Glu) and aspartic (Asp) exhibit a “umami” taste, while the sweet amino acids are alanine (Ala), glycine (Gly), serine (Ser), Thr, proline (Pro), and Lys [4]. Arginine (Arg), histidine (His), Leu, Ile, methionine (Met), Phe, Try, tyrosine (Tyr), and Val can bring about a bitter taste [5].

A high-salinity environment is normally used in soy sauce fermentation for generating a unique flavor and preventing microbial growth [6]. Typically, the concentration of sodium chloride in soy sauce is around 18–20 *w*/*v* % [7]. It is known that excessive intake of high-sodium food poses a severe risk for human health, causing hypertension, kidney disease, and other series of diseases. As per WHO recommendations, the daily sodium consumption should be less than 2 g (equivalent to 5 g NaCl). A previous study showed that sodium chloride content between 5 and 10% (*w*/*v*) in soy sauce can benefit human health without compromising taste [8]. 

Several techniques such as electrodialysis (ED) [9,10], ion exchange resin [11,12], freeze concentration [13], and nanofiltration [14] have been claimed as effective approaches to remove sodium chloride from soy sauce. Freezing concentration can effectively remove salt and preserve the taste, but its application is hindered by the complexity of the technology [13]. Nanofiltration can effectively improve the nutrient retention in the process of soy sauce desalination [13], but this process consumes a large amount of energy (5.7 kWh/kg) [14], and membrane fouling requires a large amount of water cleaning; therefore, this technology is not suitable for soy sauce desalination [15,16]. ED, as a promising technology, has not only been widely used in industrial applications such as desalination, water and wastewater treatment, food and pharmaceutical production, and other separation and purification processes, but also can be combined with other processes such as adsorption and nanofiltration to improve overall desalination efficiency [15,16]. ED is an electric-driven process in which ions and charged compounds are separated while uncharged molecules are retained in the feed [17]. Studies showed that ED can be used for desalination [18] and functional compound fractionation (such as a peptides, amino acids, proteins) [19] in the food and pharmaceutical industries. 

In recent years, various investigations have been reported on the desalination of soy sauce by the ED process. The salt removal of up to 75% was achieved without changing the flavor, color, and taste [8]. Fidaleo [20] applied a mathematical model in the soy sauce desalination with ED and reported a 30% loss in amino nitrogen. Amino acid separation using ED has also been studied for its biorefinery applications [9,21]. However, to our knowledge, there has not been a study focusing on the individual amino acid transport across the anion exchange membrane during soy sauce desalination with ED nor its relationship with the flavor of soy sauce. Understanding the transport mechanism can benefit the optimization of the operational procedure for soy sauce desalination and the improvement of membrane design. 

Therefore, in this study, the effect of current density and pH on the desalination rate, current efficiency, and amino acid lost rate during the soy sauce desalination process was investigated. The transport mechanism of different amino acids was related to their molecular weight, initial concentration, and molecular configuration. A set of operational strategies was proposed in order to achieve optimal salt removal and amino acid retention.

## 2. Materials and Methods

### 2.1. Materials

Raw soy sauce was provided by Qingdao Dengta Flavoring and Food Co., Ltd., Qingdao, China, with NaCl concentration 180 g/L and pH 5. Chemicals used in the experiments were NaCl (99.5%), NaOH anhydrous (96–99%), H_2_SO_4_ (95–98%), and Na_2_SO_4_ anhydrous (99%). All the reagents were purchased from Sinopharm Chemical Reagent Co. Ltd. (Shanghai, China). Commercial standard anion exchange membranes (CJMA) and standard cation exchange membranes (CJMC) were bought from ChemJoy Membrane Co., Ltd., Hefei, China. The physical and chemical properties of the cation and ion exchange membrane used in the experiment are shown in Table 1. These IMEs were fabricated with polyphenylene oxide and were stable at our operational conditions. The membranes have been used in the brine desalination process [22]. 

### 2.2. Amino Acids in the Soy Sauce

The fermentation process of soy sauce production took around 6 months at room temperature [23]. During the process, various amino acids, organic fatty acids, esters, and other flavor substances were produced from microbial activities, and this formed the unique aroma and flavor of soy sauce. Although the substances with different aromas and flavors are worth studying, this investigation mainly focused on amino acids, which may migrate through ion exchange membranes by the electric field. As analyzed, the concentration of the main amino acids in the soy sauce is shown in Table 2. Among them, six amino acids (Lys, Phe, Met, Thr, Ile, and Leu) are essential to the human body, while all the amino acids in this table are referred to in terms of three kinds of taste: sweet, umami, and bitter [24]. 

It can be found in Table 2 that some bitter-flavored amino acids also exhibited considerably high concentration: Leu 6.989 mg/mL, Arg 5.216 mg/mL, and Ile 5.032 mg/mL, while all the sweet amino acids showed a moderate concentration from 2.013 to 4.234 mg/mL. 

### 2.3. ED Setup

**Figure 1:**
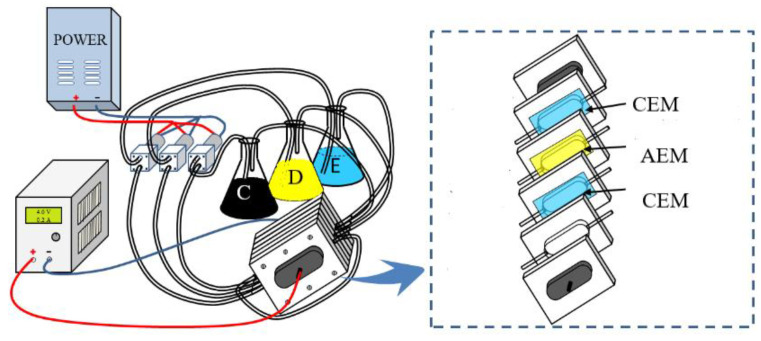
ED setup for soy sauce desalination. D—diluate vessel, C—concentrate vessel, E—electrode rinsing solution vessel, CEM—cation exchange membrane, AEM—anion exchange membrane.

A schematic diagram of the ED apparatus (custom-made) used in this investigation is shown in Figure 1. The ED stack consisted of one diluate compartment, one concentrate compartment, and two electrode rising compartments on each side. These compartments were separated by three membranes (one anion exchange membrane and two cation exchange membranes). All flow compartments had volumes of 132 cm^3^ (13.2 cm × 10 cm × 1 cm). The membrane active surface area was 20 cm^2^, and the intermembrane distance was 1 cm. Anode and cathode were made of titanium coated with ruthenium. The flow rate of all streams was 1.12 cm/s. 

During the experiments, the volumes of diluate, concentrate, and electrode rinsing solution were all 300 mL. At the beginning of the experiment, the diluate compartment was recirculated with the soy sauce and the concentrate compartment was pumped with 0.1 M NaCl solution. During the ED process, the soy sauce was diluted and the concentration of the NaCl solution was increased. The electrodes were rinsed by recirculating an aqueous solution containing 0.1 M Na_2_SO_4_ for all experiments. The flow rates of diluate, concentrate, and electrode rinsing solution recirculation were all set as 1.12 cm/s, which was manually regulated using ball valves of the flow meters. The current density was kept constant using a direct current power source HSPY-60–05 (Hanshengpuyuan, China) between the two working electrodes during each experiment. Scanning electron microscopy (SEM) technique was used to qualitatively characterize the foulant layer on fouled AEM samples. Pristine and fouled membrane samples were prepared before SEM imaging by coating with a thin layer of gold to reduce membrane surface charge. SEM images of the membrane surface were taken at a voltage of 5 kV using the Hitachi 8200 (Tokyo, Japan). Atomic force microscope (AFM) used the Park systems NX-Wafer.( Santa Clara, CA, USA) Moreover, energy-dispersive X–Ray spectroscopy (EDS) was used to quantitatively determine the atom composition on the pristine and fouled AEM samples at an approximate penetration depth of 2–5 μm, using the IXRF (Austin, TX, USA). 

Each experiment ran for 10 h, and 1 mL sample solution was taken every 2 h from the concentrate vessel and the diluate vessel, independently. Conductivity (range: 20–199.9 ms/cm, P902, Shanghai Youke Instrument Co., Ltd., Shanghai, China) and pH meter (P901, Shanghai Youke Instrument Co., Ltd., Shanghai, China) were used to measure the electrical conductivity and pH of the samples at each stage of the experiment. High-performance liquid chromatography (Agilent 1260, Agilent Corporation, Santa Clara, CA, USA) was used to measure the content of amino acids in soy sauce. Ion concentration was measured by ion chromatography (IC1826, SOPTOP, Shanghai, China).

In the adsorption experiment, 300 mL of soy sauce was taken and placed into two conical flasks numbered 1 and 2, in which 20 cm^2^ IEMs (the effective area of the membrane during the operation of the ED experiment) were put into the conical flask numbered 2. The environment of desalination chamber solution circulation during the operation of ED desalination was simulated under the action of a magnetic agitator, and the operation time was 10 h. The amino acid content of the final soy sauce was determined. 

### 2.4. Data Analysis

The desalination degree during the ED experiment was calculated by the following equation:

(1) $$\mathrm{Desalination}\;\mathrm{degree}\;(\%)=\frac{\mathrm{Ci}-\mathrm{Cf}}{\mathrm{Ci}}\times 100\%$$

where Ci and Cf refer to the initial and final salt concentrations, respectively. As most of the salt content in the soy sauce was NaCl [25,26], the multi-ion effect was not discussed in the study. To simplify the data analysis, we assumed the conductivity was proportional to the salt concentration. This approach was used widely in the previous studies on soy sauce desalination [27]. The relationship between conductivity and salt concentration is also plotted in Figure S1, in which the conductivity was measured at different dilutions of original soy sauce. Therefore, the desalination degree is calculated by

(2) $$\mathrm{Desalination}\;\mathrm{degree}\;(\%)=\frac{\mathrm{ki}-\mathrm{kf}}{\mathrm{ki}}\times 100\%$$

where k_i_ and k_f_ refer to initial and final salt conductivities, respectively.

The loss rate of amino acids during the desalination experiment was calculated by

(3) $$\mathrm{Amino}\;\mathrm{acid}\;\mathrm{loss}\;\mathrm{rate}\;(\%)=\frac{\left(\mathrm{Cdo}-\mathrm{Cdi}\right)}{\mathrm{Cdf}}\times 100\%$$

where C_do_ and C_di_ refer to initial and final amino acid concentrations, respectively. C_df_ is the initial concentration of total amino acids in soy sauce. The total amino acid loss rate is the total amino acid loss/initial total amino acid content.

The current efficiency of the desalting process is calculated according to the following formula:

(4) $$\eta =\frac{\mathrm{ZFV}\Delta C}{\mathrm{NIt}}\times 100\%$$

where η is the current efficiency, F is the Faraday constant (96485 C/mol), Z is the charge number of the ion, V is the final volume (L), ∆C is the concentration change in the diluate (mol/L), N is the number of cell pairs, I is the applied current (A), and t is the time (s).

The adsorption rate of amino acids by ion exchange membrane is calculated as follows:

(5) $$\mathrm{Adsorption}\;\mathrm{rate}\;\mathrm{of}\;\mathrm{amino}\;\mathrm{acids}=\frac{\left(\mathrm{Cdo}-\mathrm{Cdi}\right)}{\mathrm{Cdo}}\times 100\%$$

where C_do_ and C_di_ refer to initial and final amino acid concentrations, respectively.

Energy consumption was an important economic index in the ED desalination experiment [28], and the calculation formula is shown as follows:

(6) $$E=\frac{\int_{0}^{t}\mathrm{UI}\mathrm{dt}}{m}$$

where E, U, I, t, and m represent the energy consumption (kWh/kg), voltage (V), current (A), time (h), and the amount of salt that moved from the diluate to the concentrate compartment (kg), respectively.

## 3. Results and Discussion

### 3.1. Desalination Degree and Amino Acid Loss Rate in the ED Process

#### 3.1.1. Effect of Current Density on Desalination Degree

**Figure 2:**
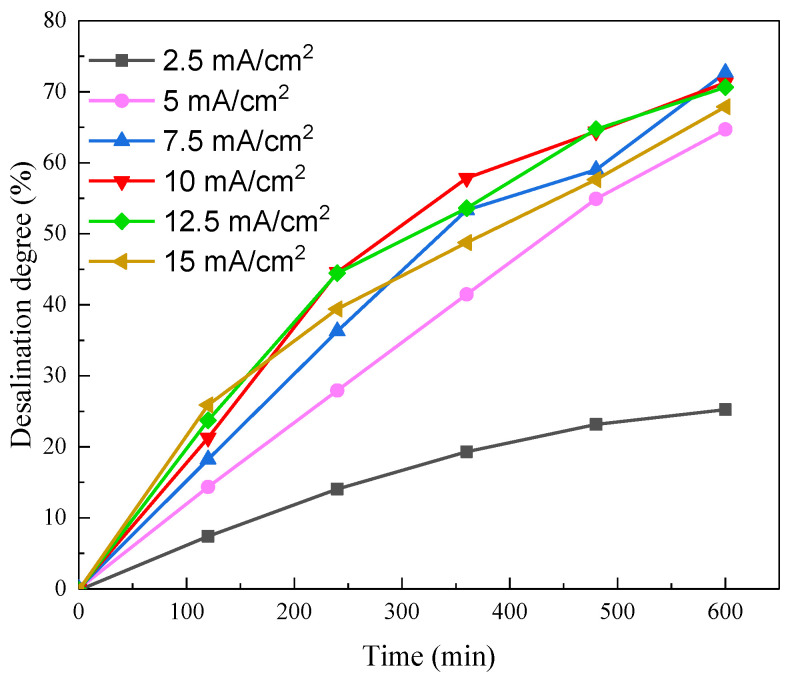
Desalination degree as a function of current density in the soy sauce desalination experiments.

To optimize the applied current, we selected values of 2.5, 5, 7.5, 10, 12.5, and 15 mA/cm^2^ for this investigation. As can be seen from Figure 2, the desalination degree at the end of each test increased from 22.7% to 64% as the current density increased from 2.5 mA/cm^2^ to 5 mA/cm^2^, and slightly dropped when the current density further increased to 15 mA/cm^2^. This final desalination degree ranged from 64% to 70% when the current density was between 5 and 15 mA/cm^2^. The desalination degree was limited below 70%, and this was likely due to the inevitable electro-osmotic water transport that was dragged by the movement of the salt ions. The water flux correlated strongly with the current density, as shown in Figure S1. 

**Figure 3:**
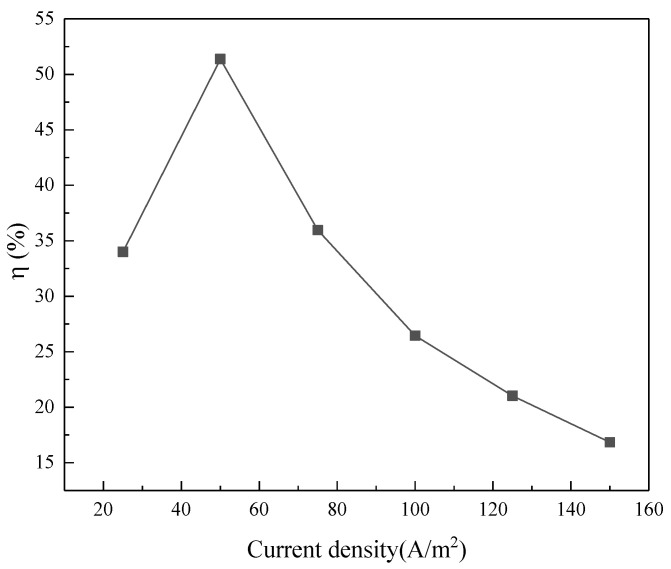
Correlation between current efficiency and current density for the soy sauce desalination process.

To further understand the desalination process, we calculated the current efficiency as a function of the applied current density, which is shown in Figure 3. It can be found that the current efficiencies were 34% and 52%, respectively, when the current densities of 2.5 and 5 mA/cm^2^ were used. When the current density was higher than 5 mA/cm^2^, the current efficiency decreased rapidly with the increased current density. This observation was unsurprising and was found in the previous studies [29,30,31]. The voltage as a function of time at different current densities was shown in Figure S2. The reduction of current efficiency may be explained by two aspects: (1) The limiting current was reached after increasing the applied current density above 5 mA/cm^2^. This limiting current can hardly be quantified due to the dynamic change of the desalination process. In this case, part of the current (or energy) was used for desalination and the rest could be consumed by water splitting and electro-convection [32]. The calibration curve for conductivity to concentration is now provided in Figure S3.Water splitting was confirmed by the decrease of pH in the concentrate compartment from 6.9 to 5.0, as shown in Figure S4. The pH in the diluate compartment remained unchanged as the amino acids in the soy sauce functioned as a pH buffer. (2) Some potential membrane fouling such as charged small proteins, peptides, and colloids potentially forming a gel layer on the membrane surface led to the membrane fouling tendency increasing when a higher current density was applied, hence elevating the electric resistance of the ED stack. Combined with the above two reasons, when the current was greater than 5 mA/cm^2^, the electric energy used in the soy sauce desalination process was not only for desalination, but also consumed in other aspects (electro-convection, water splitting, and generating heat, as addressed above). The limiting current was tested at the beginning of the experiment as shown in the Figure S5. Therefore, the total energy consumption increased with the increase of the current, while the amount of removed salt did not increase proportionally. On the basis of the results, we believe that 5 mA/cm^2^ of current density is an appropriate value for further experiments.

#### 3.1.2. Effect of pH on Desalination Degree and Amino Acid Loss Rate

**Figure 4:**
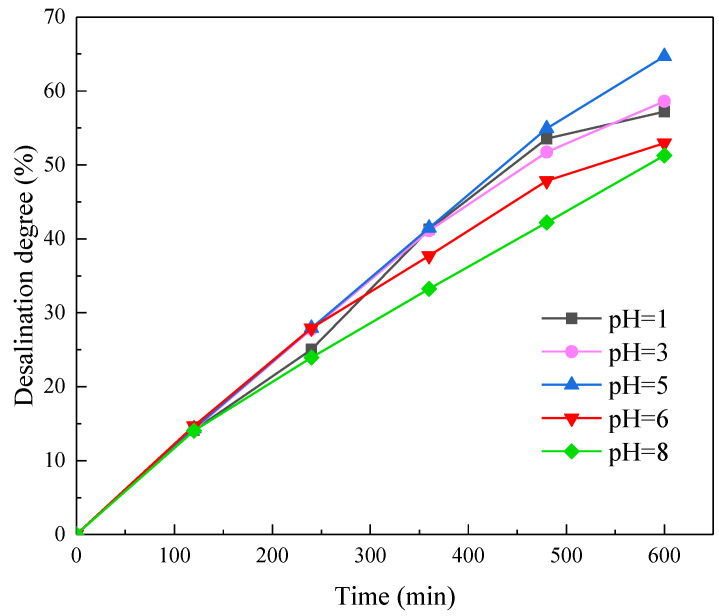
Desalination degree under different pH values.

**Figure 5:**
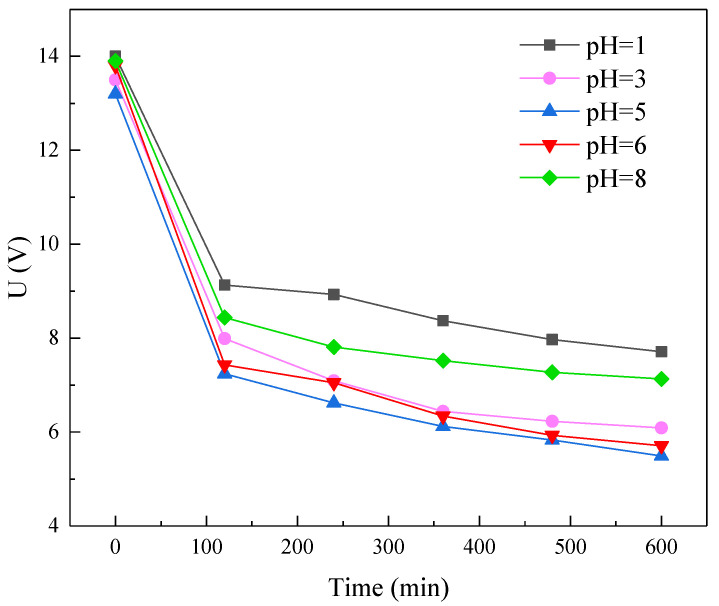
Cell voltage as a function of time measured at soy sauce pHs of 1, 3, 5, 6, and 8 at a current density of 5 mA/cm^2^.

To study the effect of pH value on desalination degree and amino acid loss rate, we adjusted the pH values of the diluate (soy sauce) to 1, 3, 5, 6, and 8. ED desalination was carried out under these conditions, and the results are shown in Figure 4. It can be observed that the desalination degree had a weak correlation with the diluate pH: when the initial pH was 5, the results showed the highest desalination degree of 64% at the end of the experiment, while when pH values were 6 and 8, the desalination degree was around 56%. The lowest desalination degree (around 51%) was obtained when the initial soy sauce was adjusted to a more acidic condition (pH 1 and 3). The variation of desalination degree as a function of pH may be possibly attributable to the fact that 5 was the original pH of the soy sauce and was a stable condition for the colloids inside. The desalination degree over time was plotted in the following Figure S6. However, when the pH becomes more acidic or alkaline, the colloid in the soy sauce may accumulate and contaminate the membrane surface, leading to an increased membrane resistance and cell voltage. This was demonstrated by the voltage–time profile, as shown in Figure 5. The initial salt concentration gradient over the membrane was the highest. At the beginning of the experiment, the ions were transported quickly through the membranes, resulting in a rapid decrease of resistance and cell voltage. The decrease of voltage after 100 min became gradual as the gradient slowly decreased. Finally, the neutral pH led to the lowest voltage compared to the acidic or alkaline solution [33]. 

**Figure 6:**
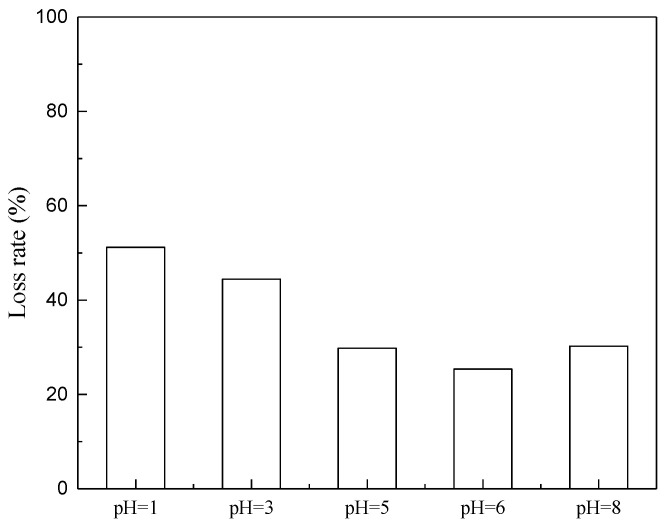
Amino acid loss rate at different pH values.

On the other hand, the pH of the soy sauce also influenced the retention of amino acids. At a specific pH, the amine and carboxyl groups exhibited the same degree of ionization, and the overall net charge of the molecule was zero [34]. It is desirable to control the loss of amino acids by adjusting the pH of the soy sauce to the pI of those major amino acids. As seen from Figure 6, when pH values were more acidic (1 and 3), the loss rate of amino acids in soy sauce showed relatively higher values (50% and 45%, respectively). Oppositely, when pH was 6, the loss rate exhibited the lowest value (around 25%). When the pH of soy sauce was 5, the amino acid loss rate was 29.8%. This was because most of the amino acids in soy sauce are pI between 5 and 6. This was shown in Table 3. The weighted average isoelectric point of amino acids in soy sauce was 5.71, and therefore the amino acid loss rate was the lowest between pH 5 and 6. 

The average isoelectric point (pI_s_) is calculated as follows:

pI_s_=α_1_pI_1_+ ┈+α_n_pI_n_ (7)

where subscript a represents the mole fraction of each amino acid and n is the number of the amino acid present in the soy sauce.

Combined with desalination degree analysis, pH 5 should be the optimal value for desalination of soy sauce. Therefore, given the desalination degree and the amino acid loss rate, pH 5 should be the optimized value for soy sauce desalination. According to the relevant literature [35], the energy consumption of nanofiltration is 5.7 kWh/kg, while the energy consumption of ED operation calculated by Equation (6) is 1.94 kWh/kg; the unit energy consumption of ED is much lower than that of nanofiltration. Once our process is implemented on a large scale, more sophisticated energy analysis approaches such as standard primary energy method can be considered [36]. According to the relevant literature [8], when the soy sauce is diluted to the same salt content as that treated with ED, the experimental personnel cannot distinguish the taste, color, and smell, that is, the soy sauce treated with ED is similar to that treated with diluted soy sauce, and therefore ED is more suitable for soy sauce desalination. The total loss rate of amino acids at different pH values is shown in Figure 6. The highest loss rate was found at pH 1, and the lowest at pH 6. When the pH value of soy sauce was adjusted to 5, the loss rate of total amino acids in soy sauce was still relatively low. Considering the highest desalination degree was obtained at pH 5, we explored the transport mechanism using soy sauce at pH 5.

### 3.2. Transport Mechanism of the Amino Acids in the ED Process

#### 3.2.1. Electro-Transport of Amino Acids in the ED Process

**Figure 7:**
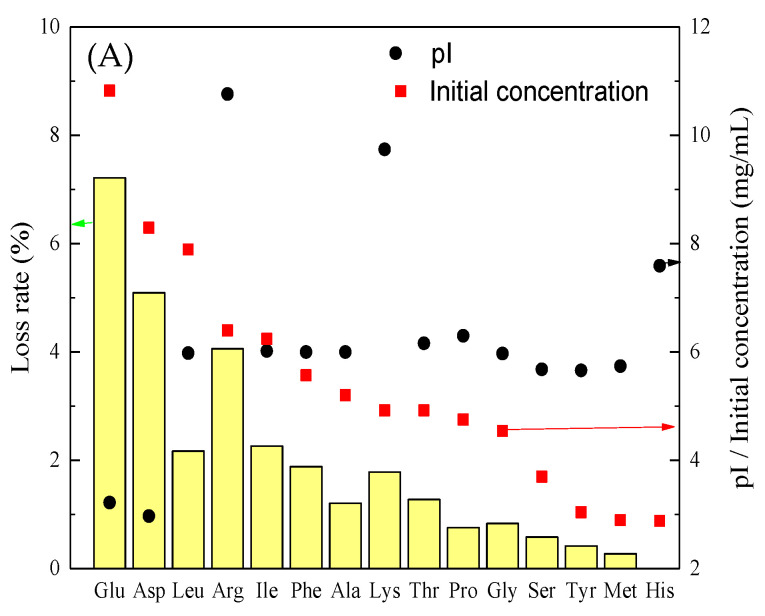

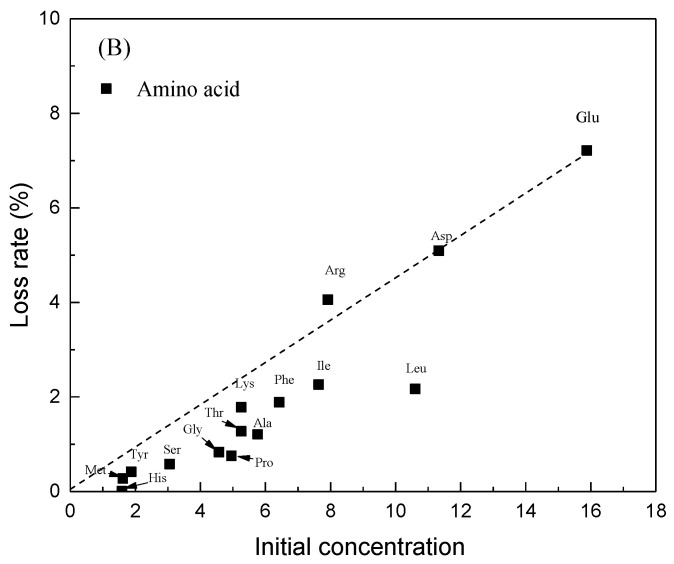
(**A**) Overview of the loss rate of amino acids, isoelectric point, and their initial concentration during the desalination process at pH = 5. (**B**) The relationship between the loss rate of amino acids and their initial concentration. pI: isoelectric point.

To further understand the mechanism of amino acid migration in the ED process, we analyzed the loss rate of amino acids under the optimal desalination conditions (current density of 5 mA/cm^2^, pH of 5), which is shown in Figure 7A. It can be found that the loss rate of amino acids in soy sauce was related to both the initial concentration and the pI of amino acids. When amino acids pI < pH, the amino acid was positively charged, and when the amino acid pI > pH, the amino acid was negatively charged. In terms of the experimental conditions of Figure 7 (pH = 5), most of the amino acids were negatively charged as their pI was higher than the pH. These charged amino acids can pass through AEMs and CEMs under an electric field, resulting in the loss of amino acids in soy sauce.

The loss rate of amino acids was also related to their initial concentration, which can be seen in Figure 7B. Glu shared the highest amino acid content (15.88%) in the soy sauce, and the loss rate was also the highest (7.21%). Followed by Asp, the content was 11.33%, and the loss rate was 5.09%. However, the contents of Arg and Lys in total amino acids were 7.9% and 5.26%, respectively, while the loss rates were 4.06% and 1.78%, respectively. This phenomenon occurred because the isoelectric point of both amino acids deviated from the pH of soy sauce greatly (Arg, PI = 10.76, Lys, PI = 9.74), which was positively charged in the solution, and thus the loss rate was high. The loss rate of Arg was higher than that of Lys. This has to do with the structure of the Arg. Arg has a functional group of guanidine. Guanidine ((NH_2_)_2_C=NH) can be regarded as a nitrogenous analogue of carbonic acid, in which the C=O group in carbonic acid is replaced by a C=NH group. Most guanidine derivatives contain salts of conjugated acids. Conjugate acids are called guanidine cations (C(NH_2_) _3+_). The symmetrical ion in this plane consists of three amino groups, each of which has a covalent bond with the central carbon atom of order 4/3, causing arginine to become positively charged in the soy sauce solution. Therefore, arginine has a high loss rate during ED [27]. Among these amino acids, His (pI = 7.59) had the lowest loss rate (0.006%) because it had the lowest content, accounting for 1.59% of the total amino acids. Another reason may be due to its aromatic structure. Bukhovets [37] investigated the principal distinction of the aromatic amino acid transport during the ED. It was reported that the loss rate of aromatic amino acids was very low. 

A linear line between the zero point and Glu was added in Figure 7B. This line provided a fair comparison of the transport rate of other amino acids with Glu. Most of the amino acids were below the line, suggesting that the transport rate of these amino acids was lower than Glu. However, Arg had a slightly higher transport rate than Glu, which could have been due to the fact that they carried different charge species. The membrane properties should be explored in future studies. A denser membrane might be needed to decrease the loss rate of amino acids in soy sauce.

#### 3.2.2. Adsorption Rate of Amino Acids in Soy Sauce by IEMs

**Figure 8:**
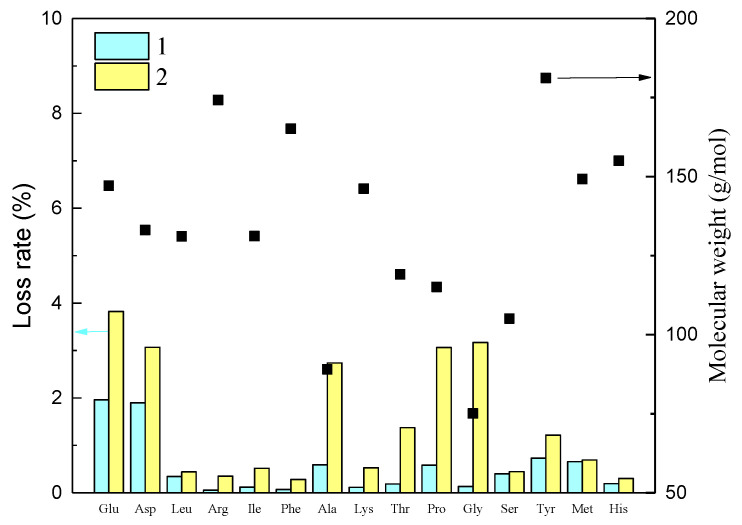
Loss rate of amino acids (black square) in the flask: 1 represents the data without membrane, 2 represents the data with membrane.

In the ED experiment, besides the desalination rate of soy sauce and amino acid loss rate, the adsorption of amino acids to IEMs might also have been an important factor affecting the loss rate. As shown in Figure 8, the content of amino acids in the conical flask soy sauce was lost, but the loss rate was only between 0.06% and 1.96. The amino acids could either be attached on the surface of the conical flask or degraded during the long-term stirring process. Among them, the proportions of Glu and Ala in the total amino acids were 15.88 % and 11.33 %, respectively, which showed a higher loss rate (1.96% and 1.9%, respectively). The loss rate of amino acid with the membrane (no. 2) was remarkably higher than that without membrane (no. 1), especially for those with low molecular weight. More specifically, the molecular weight of Gly was 75.07 Da, and the adsorption rates of amino acids had the largest difference before and after adding IEMs, which were 0.133% and 3.17%, respectively. The molecular weight of Ala was 89.09 Da, and the differences in amino acid adsorption rates before and after adding IEMs were 0.59% and 2.74%. This was because the low-molecular-weight amino acids had a higher affinity to the membrane. On the other hand, as shown in this figure, the loss rate of Pro also exhibited considerable differences, being 0.58 and 3.06, respectively. This may have been caused by the aromatic nature of Pro. Tanaka et al. [38] reported that the affinity between aromatic organics and membrane was higher than that of aliphatic organics to the membrane. Therefore, this experiment confirmed that both the size and structure of amino acids influenced the membrane adsorption. The charge state of amino acids in the adsorption experiment is shown in Table S1.

### 3.3. Analysis of Pristine and Fouled IEMs

**Figure 9:**
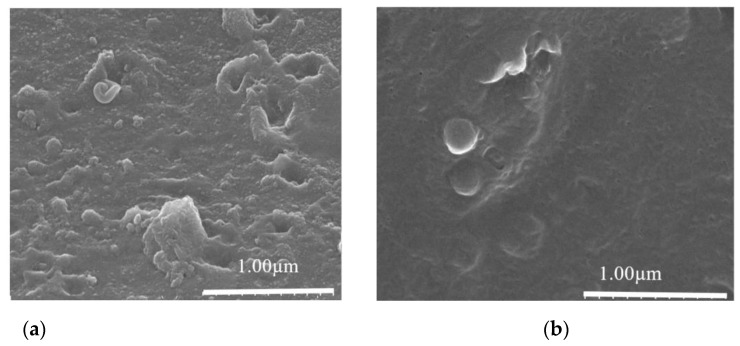

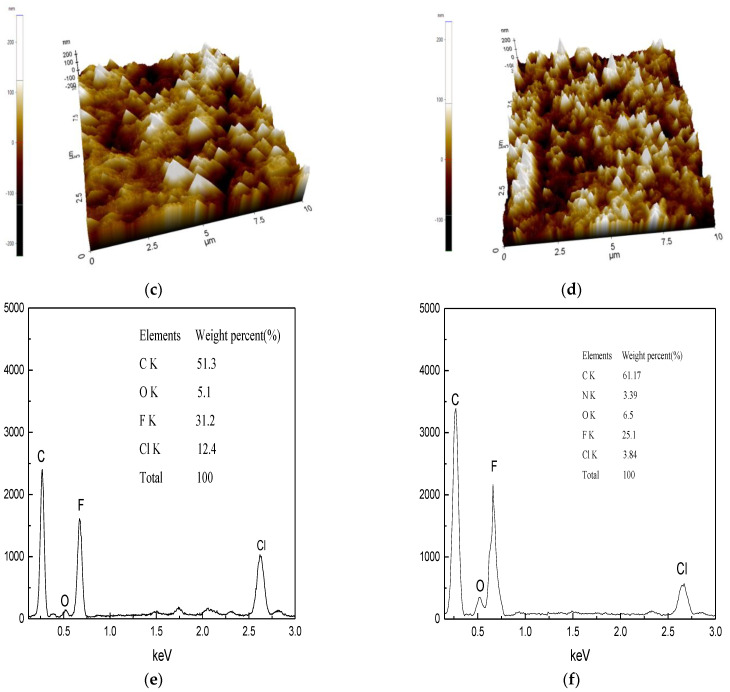
Images of SEM (**a**,**b**), AFM (**c**,**d**), and EDS (**e**,**f**) of the pristine and fouled IEMs.

Finally, SEM, AFM, and EDS were employed to characterize the pristine and fouled ion exchange membranes. As shown in the SEM pictures (Figure 9A,B), compared with the pristine membrane (Figure. 9A), the surface of the membrane (Figure 9B) was covered with foulants. As analyzed in Figure 9 C (pristine) and D (fouled), the peaks of the membrane surfaces were 252.74 and 239.96 nm, respectively, and the roughness Ra were 46.875 and 37.02 nm, respectively. The reduction of membrane roughness after the desalination experiment suggested that a fouling layer was formed on the membrane surface, as shown in the previous study [39]. EDS analysis showed the elements detected on the membrane surface before and after the desalination process (Figure 9E,F). It was worth noting that the number of carbon atoms increased from 51.314% (pristine membrane, Figure 9E) to 61.17% (fouled membrane, Figure 9F), indicating again the formation of a fouling layer. In terms of SEM, AFM, and EDS, it was confirmed that the membrane was fouled by some compositions in the soy sauce during desalination. However, the compositions should be further analyzed, and strategies have to be taken to reduce membrane fouling and hence improve the energy efficiency.

## 4. Conclusions

This work investigated the desalination of soy sauce by ED for various factors on desalination degree and amino acid loss. The effect of current density and initial pH on the desalination degree and amino acid loss rate was studied by single-factor experiments. The optimum operating condition was determined for this process, with the desalination degree of 64%, the total amino acids loss rate of 29.8% (sum of the loss rate of each amino acid), and the energy consumption of 1.94 kWh/kg, at the current density of 5 mA/cm^2^ and pH of 5.

The desalination degree increased with the increase of current; however, when the current density was greater than 5 mA/cm^2^, the current efficiency decreased. Furthermore, it was found that when the pH of soy sauce was between 5 and 6 (close to the pIs of most amino acids), the amino acid loss rate was the lowest. In this study, it was also demonstrated that the molar weight and structure of amino acids affected their transport behavior in the ED and the membrane adsorption rate. Amino acid with smaller molar weight migrated faster through IEMs than those of larger ones. The loss rate of aromatic amino acids was lower than those of non-aromatic amino acids, even though they had a similar molecular weight. Finally, SEM, AFM, and EDS analyses illustrated that the membrane was fouled by soy sauce during desalination. Therefore, further studies could focus on mitigating membrane fouling in the soy sauce desalination. 

## Figures and Tables

**Table 1:** Properties of the pristine IEMs used in the experiments.

Type	Resistance (Ω/Cm^2^)	Water Content (%)	Ion Exchange Capacity(Mmol/G)	Selective Transmittance	FunctionalGroup	Thickness(Mm)
CJMC	2.5–3.5	40–50	0.8–1.0	>0.93	–SO_3_Na	0.18–0.21
CJMA	3.5–4.5	15–20	0.5–0.6	>0.93	–NR_4_Cl	0.14–0.16

**Table 2:** Composition, concentration, and properties of amino acids in the soy sauce.

Amino Acids	Concentration (mg/mL)	Isoelectric Point (pI)	Molecular Weight (Da)	Taste
Glutamate (Glu)	10.465	3.22	147.13	Umami
Aspartate (Asp)	7.465	2.97	133.09	Umami
Leucine (Leu)	6.989	5.98	131.1	Bitter
Arginine (Arg)	5.216	10.76	174.2	Bitter
Isoleucine (Ile)	5.032	6.02	131.17	Bitter
Phenylalanine (Phe)	4.234	5.49	165.19	Sweet
Alanine (Ala)	3.798	6	89.09	Sweet
Lysine (Lys)	3.465	9.74	146.19	Sweet
Threonine (Thr)	3.465	6.16	119.12	Sweet
Proline (Pro)	3.265	6.3	115.13	Sweet
Glycine (Gly)	3.013	5.97	75.07	Sweet
Serine (Ser)	2.013	5.68	105.09	Sweet
Tyrosine (Tyr)	1.234	5.64	181.19	Bitter
Methionine (Met)	1.065	5.74	149.21	Bitter
Histidine (His)	1.046	7.59	155	Bitter

**Table 3:** The weighted average of amino acid isoelectric points.

Amino Acids	Concentration (mg/mL)	Molar Fraction of Total Amino Acids (%)	Isoelectric Point (pI)
Glutamate (Glu)	10.465	14.88	3.22
Aspartate (Asp)	7.465	11.73	2.97
Leucine (Leu)	6.989	11.15	5.98
Arginine (Arg)	5.216	6.26	10.76
Isoleucine (Ile)	5.032	8.02	6.02
Phenylalanine (Phe)	4.234	5.36	5.49
Alanine (Ala)	3.798	8.91	6
Lysine (Lys)	3.465	4.96	9.74
Threonine (Thr)	3.465	6.09	6.16
Proline (Pro)	3.265	5.93	6.3
Glycine (Gly)	3.013	8.4	5.97
Serine (Ser)	2.013	4	5.68
Tyrosine (Tyr)	1.234	1.4	5.64
Methionine (Met)	1.065	1.49	5.74
Histidine (His)	1.046	1.42	7.59
The total amino acid	61.765	100	5.71 *

* This was the calculated average isoelectric point.

## Data Availability

The data that support the findings of this study are available from the corresponding authors upon reasonable request.

## Supplementary Materials

The following are available online at https://www.mdpi.com/article/10.3390/membranes11060408/s1,: Figure S1. (A) Diluate volume change as a function of time and (B) the correlation between water flux and current density. Figure S2. (A) Voltage changes as a function of time at the current density of 25, 50, 75, 100, 125, and 150 A/m2. (B) The relationship between voltage measured at the end of the experiment and the applied current density. Figure S3. The correlation between the conductivity and the NaCl concentration. The pH change in the concentrate compartment during the desalination process. Figure S4. The pH change in the concentrate compartment during the desalination process. Figure S5. Reverse current density and resistance diagram. Figure S6. Desalination degree as a function of current density in the soy sauce desalination ex-periments. Table S1. Charge state of amino acids.

## References

[1] Ta Y.W., Mun S.K., Lee F.S., Lithnes K.P., Wu T.Y., Kan M.S., Siow L.F., Palni L.K. (2010). Effect of temperature on moromi fermentation of soy sauce with intermittent aeration. Afr. J. Biotechnol..

[2] Kim J.-S., Lee Y.-S. (2007). A study of chemical characteristics of soy sauce and mixed soy sauce: Chemical characteristics of soy sauce. Eur. Food Res. Technol..

[3] Sugawara T., Saraprug D., Sakamoto K. (2019). Soy sauce increased the oxidative stress tolerance of nematode via p38 MAPK pathway. Biosci. Biotechnol. Biochem..

[4] Zhang Y.F., Tao W.Y. (2009). Flavor and taste compounds analysis in Chinese solid fermented soy sauce. Afr. J. Biotechnol..

[5] Devanthi P.V.P., Gkatzionis K. (2019). Soy sauce fermentation: Microorganisms, aroma formation, and process modification. Food Res. Int..

[6] Diez-Simon C., Eichelsheim C., Mumm R., Hall R.D. (2020). Chemical and Sensory Characteristics of Soy Sauce: A Review. J. Agric. Food Chem..

[7] Wang Q., Ying T., Jiang T., Yang D., Jahangir M.M. (2009). Demineralization of soybean oligosaccharides extract from sweet slurry by conventional electrodialysis. J. Food Eng..

[8] Fidaleo M., Moresi M., Cammaroto A., Ladrange N., Nardi R. (2012). Soy sauce desalting by electrodialysis. J. Food Eng..

[9] Readi O.K., Gironès M., Nijmeijer D.C. (2013). Separation of complex mixtures of amino acids for biorefinery applications using electrodialysis. J. Membr. Sci..

[10] Hidalgo A., Murcia M. (2021). Membranes for Water and Wastewater Treatment. Membranes.

[11] Suwal S., Doyen A., Bazinet L. (2015). Characterization of protein, peptide and amino acid fouling on ion-exchange and filtration membranes: Review of current and recently developed methods. J. Membr. Sci..

[12] Stenina I., Yaroslavtsev A. (2021). Ionic Mobility in Ion-Exchange Membranes. Membranes.

[13] Watanabe M., Tesaki S., Arai S. (1996). Production of Low-salt Soy Sauce with Enriched Flavor by Freeze Concentration Using Bacterial Ice Nucleation Activity. Biosci. Biotechnol. Biochem..

[14] Luo J., Ding L., Chen X., Wan Y. (2009). Desalination of soy sauce by nanofiltration. Sep. Purif. Technol..

[15] Zhang Y., Van der Bruggen B., Pinoy L., Meesschaert B. (2009). Separation of nutrient ions and organic compounds from salts in RO concentrates by standard and monovalent selective ion-exchange membranes used in electrodialysis. J. Membr. Sci..

[16] Nh K.C., Thu K., Shahzad M.W., Chun W. (2014). Progress of adsorption cycle and its hybrids with conventional multi-effect desalination processes. IDA J. Desalination Water Reuse.

[17] Moscetti R., Massantini R., Fidaleo M. (2019). Application on-line NIR spectroscopy and other process analytical technology tools to the characterization of soy sauce desalting by electrodialysis. J. Food Eng..

[18] Zhang Y., Pinoy L., Meesschaert B., Van Der Bruggen B. (2011). Separation of small organic ions from salts by ion-exchange membrane in electrodialysis. Aiche J..

[19] Wei Q., Wang H., Chen Z., Lv Z., Xie Y., Lu F. (2013). Profiling of dynamic changes in the microbial community during the soy sauce fermentation process. Appl. Microbiol. Biotechnol..

[20] Fidaleo M., Moresi M., Cammaroto A., Ladrange N., Nardi R. (2012). Modelling of Soy Sauce Desalting by Electrodialysis. Food Bioprocess Technol..

[21] Readi O.K., Mengers H., Wiratha W., Wessling M., Nijmeijer K. (2011). On the isolation of single acidic amino acids for biorefinery applications using electrodialysis. J. Membr. Sci..

[22] Sarapulova V., Pismenskaya N., Butylskii D., Titorova V., Wang Y., Xu T., Zhang Y., Nikonenko V. (2020). Transport and Electrochemical Characteristics of CJMCED Homogeneous Cation Exchange Membranes in Sodium Chloride, Calcium Chloride, and Sodium Sulfate Solutions. Membranes.

[23] Syifaa A.S., Jinap S., Sanny M., Khatib A. (2016). Chemical Profiling of Different Types of Soy Sauce and the Relationship with its Sensory Attributes. J. Food Qual..

[24] Ganguly A., Pang L., Duong V.-K., Lee A., Schoniger H., Varady E., Dahanukar A. (2017). A Molecular and Cellular Context-Dependent Role for Ir76b in Detection of Amino Acid Taste. Cell Rep..

[25] Kremer S., Mojet J., Shimojo R. (2009). Salt Reduction in Foods Using Naturally Brewed Soy Sauce. J. Food Sci..

[26] Lioe H.N., Wada K., Aoki T., Yasuda M. (2007). Chemical and sensory characteristics of low molecular weight fractions obtained from three types of Japanese soy sauce (shoyu)—Koikuchi, tamari and shiro shoyu. Food Chem..

[27] Lee Y.S., Homma S. (1991). Desalting by Electrodialysis to Measure Iron Chelating Activity of Melanoidin in Soy Sauce and Fish Sauce. Nippon. Shokuhin Kogyo Gakkaishi.

[28] Chakrabarty T., Rajesh A.M., Jasti A., Thakur A.K., Singh A., Prakash S., Kulshrestha V., Shahi V.K. (2011). Stable ion-exchange membranes for water desalination by electrodialysis. Desalination.

[29] Bunani S., Yoshizuka K., Nishihama S., Arda M., Kabay N. (2017). Application of bipolar membrane electrodialysis (BMED) for simultaneous separation and recovery of boron and lithium from aqueous solutions. Desalination.

[30] Yu Z. (2002). Determinations of Critical Limits in Ion Transport in Membrane Electrodialysis and Their Current vs. Voltage Characteristics for Ion Concentration Control in Pulp and Paper Mill Waste Stream. Ph.D. Thesis.

[31] Forgacs C., Ishibashi N., Leibovitz J., Sinkovic J., Spiegler K. (1972). Polarization at ion-exchange membranes in electrodialysis. Desalination.

[32] Ali M.B.S., Mnif A., Hamrouni B. (2018). Modelling of the limiting current density of an electrodialysis process by response surface methodology. Ionics.

[33] Chen D., Pomalaza-Ráez C. (2019). A self-cleaning piezoelectric PVDF membrane system for filtration of kaolin suspension. Sep. Purif. Technol..

[34] Sandoval-Lira J., Mondragon-Solorzano G., Lugo-Fuentes L.I., Barroso-Flores J. (2020). Accurate Estimation of pK(b) Values for Amino Groups from Surface Electrostatic Potential (V-S,V-min) Calculations: The Isoelectric Points of Amino Acids as a Case Study. J. Chem. Inf. Modeling.

[35] Zhang Y.F., Liu L., Du J., Fu R.Q., Van der Bruggen B., Zhang Y. (2017). Fracsis: Ion fractionation and metathesis by a NF-ED integrated system to improve water recovery. J. Membr. Sci..

[36] Ng K.C., Burhan M., Chen Q., Ybyraiykul D., Akhtar F.H., Kumja M., Field R.W., Shahzad M.W. (2021). A thermodynamic platform for evaluating the energy efficiency of combined power generation and desalination plants. Npj Clean Water.

[37] Bukhovets A., Savel’Eva A., Eliseeva T. (2009). Separation of amino acids mixtures containing tyrosine in electromembrane system. Desalination.

[38] Tanaka N., Nagase M., Higa M. (2012). Organic fouling behavior of commercially available hydrocarbon-based anion-exchange membranes by various organic-fouling substances. Desalination.

[39] Berkessa Y.W., Lang Q., Yan B., Kuang S., Mao D., Shu L., Zhang Y. (2019). Anion exchange membrane organic fouling and mitigation in salt valorization process from high salinity textile wastewater by bipolar membrane electrodialysis. Desalination.

