# Supplementary material for: Transport of Amino Acids in Soy Sauce Desalination Process by Electrodialysis
Source: Membranes (Basel). 2021 May 29;11(6):408. doi: 10.3390/membranes11060408 (PMC8228157; doi:10.3390/membranes11060408)
Supplement: Supplementary file 1 [file membranes-11-00408-s001.zip › membranes-1208001-supplementary.pdf]

membranes

Supplementary Materials

Transport of Amino Acids in Soy Sauce Desalination Process by Electrodialysis

Man Wang [1], Shaoping Kuang [1,*], Xitong Wang [1],Daihao Kang [1], Debin Mao [2], Guanlan Qian [3], Xiaodan Cai [3], Ming Tan [1,*], Fei Liu[1], Yang Zhang [1]

[1] College of Environment and Safety Engineering, Qingdao University of Science and Technology, 53 Zhengzhou Road, 266042 Qingdao, China. 17865305301@163.com (M.W.); wangxitongg@163.com (X.W.) kangdaihao@126.com (D.K.); fliu@qust.edu.cn (F.L.); zhangyang@qust.edu.cn (Y.Z.)

[2] Flemish Institute for Technological Research (VITO), Boeretang 200, BE-2400, Mol, Belgium debin.mao@vito.be

[3] Qingdao Dengta Flavoring and Food Co., Ltd., 65 Huangtai Road, 266012 Qingdao, China, qianguanlan@dengtatwp.com (G.Q.); caixiaodan@dengtatwp.com (X.C.)

* Correspondence: tanming@qust.edu.cn (M.T.); kuangshaopaoping@126.com (S.K.)

1.The volume change and the water flux were plotted and added in the supporting information (Fig. S1).

The water flux and current density had a strong linear correlation, indicating the water transport was mainly due to the electro-osmosis in which the transport of salt ions dragged the water molecule across the membrane. This inevitable water transport had a large impact on the desalination process, in which the desalination degree slightly dropped after the current density increased beyond 75 A/m^2.

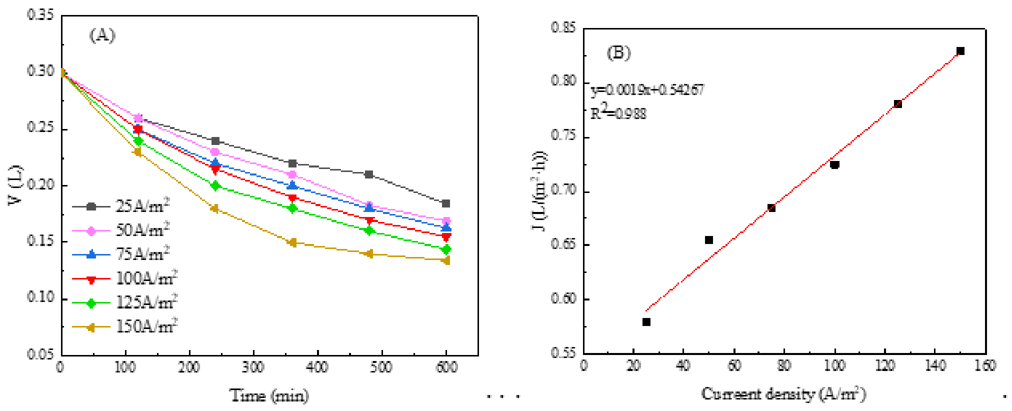

Figure S1. Diluate volume change as a function of time (**A**) and the correlation between water flux and current density (**B**).

2.The voltage as a function of time at different current densities was shown in Fig. S2A.

The voltage decreased significantly during the first 4 h in all the tests. This attributes to a decrease of cell resistance as the salt ions continuously moved to the dilute stream (0.1 M NaCl). The decrease of the voltage became gradual after 4 h due to the balanced concentration gradient. It is worth noting in Fig. S2B that the final voltage was strongly correlated with the applied current density. A voltage of 4.5 V was found at current density of 0 (open circuit condition). This suggests that the voltage (4.5V) was contributed to the redox reactions at the electrodes.

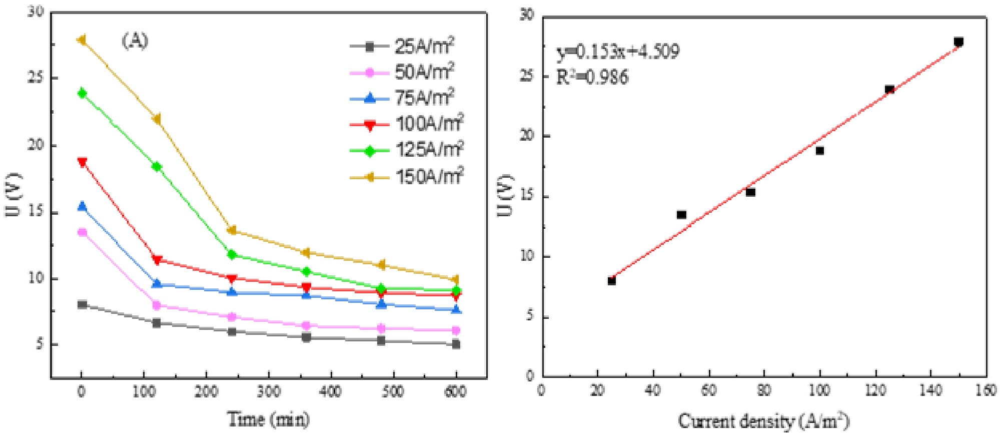

Figure S2. (**A**) Voltage changes as a function of time at the current density of 25, 50, 75, 100, 125, and 150 A/m². (**B**) The relationship between voltage measured at the end of the experiment and the applied current density.

3.The range of the conductivity probe was 20-199.9 ms/cm. The calibration curve for conductivity to concentration is now provided in the supporting information (Fig. S3).

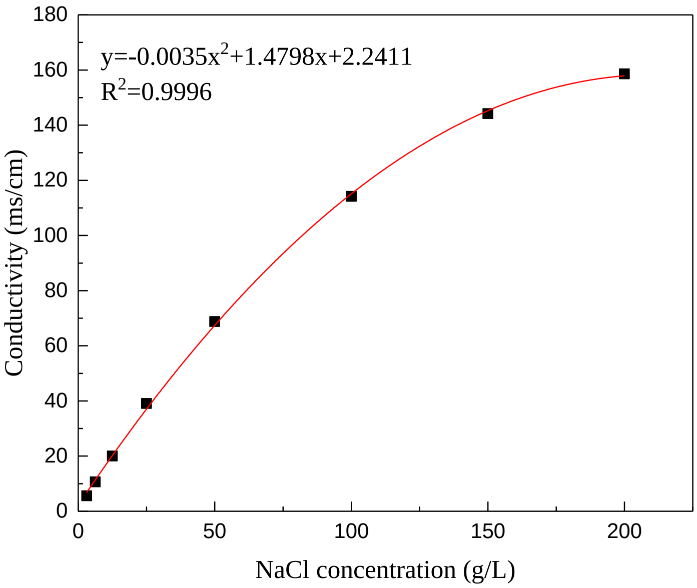

Figure S3. The correlation between the conductivity and the NaCl concentration.

4.The reduction of current efficiency may be explained from two aspects: 1, the limiting current was reached after increasing the applied current density above 50 A/m². In this case, part of the current (or energy) was used for desalination and the rest could be consumed by water splitting and electro-convection. Water splitting was confirmed by the decrease of pH in the concentrate compartment from 6.9 to 5.0, as shown in Fig. S3. The pH in the diluate compartment remined unchanged as the amino acids in the soy sauce functioned as a pH buffer.

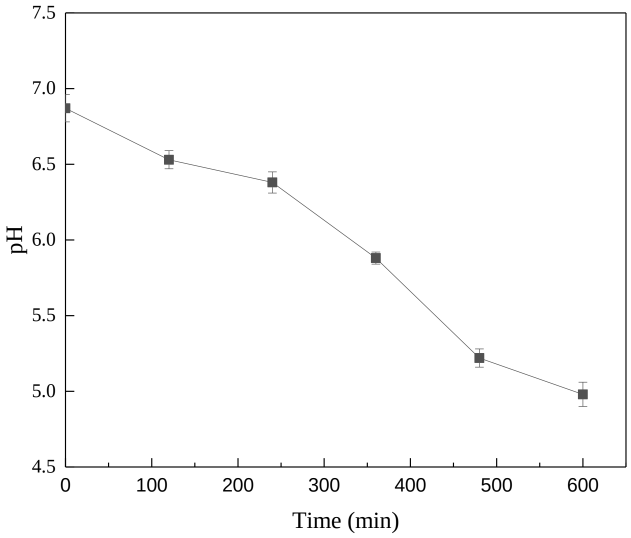

Figure S4. The pH change in the concentrate compartment during the desalination process.

5.Determination of limiting current

The limiting current was tested at the beginning of the experiment as shown in the figure S5.

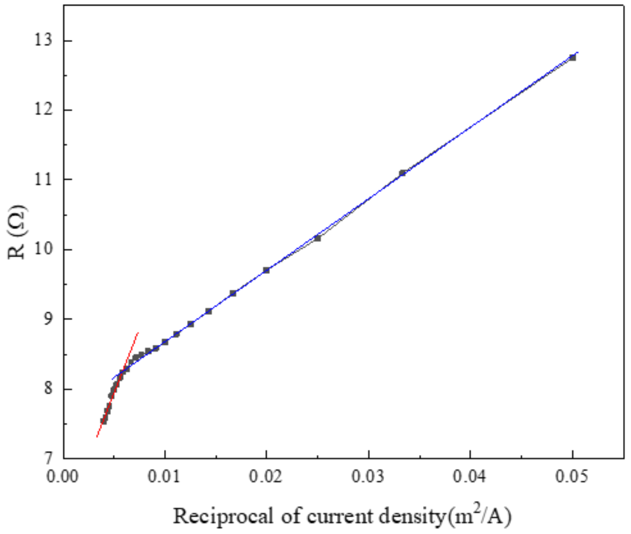

Figure S5. Reverse current density and resistance diagram.

The reciprocal curves of total ED resistance and current density are divided into two distinct regions. In current density small 0.006 m^2/A, the reciprocal of resistance is less than 8.3 Ω, appeared before the polarization phenomenon, the membrane resistance changes slowly, proved that the areas are less than the limit current obey ohm's law. When the current is further increased, until it exceeds the limit current of the IEMs. At this point, ions in the boundary layer of the diluate compartment quickly cross the membrane surface, while ions in the solution have no time to migrate through the boundary layer, resulting in a sharp drop in the ion concentration in the boundary layer. Therefore, the membrane resistance of the IEMs increases. In order to protect the ED equipment, take 80% of its limit current [1], i.e. 3A, as the maximum working current, In practical operation, the limit current may appear in advance due to membrane surface fouled and resistance increase when the limit current runs with ED for a long time.

6. A replicate test was performed for several selected current densities (i.e., 5, 10, and 15 mA/cm2). The desalination degree over time was plotted in the following figure. As can be seen, the data variation was rather small.

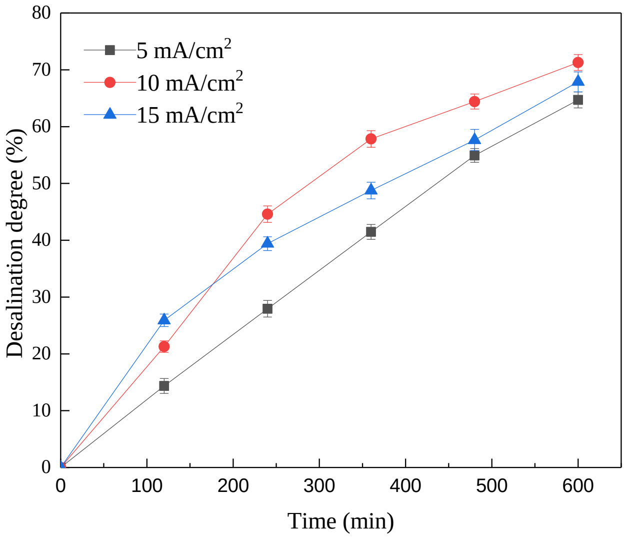

Figure S6. Desalination degree as a function of current density in the soy sauce desalination experiments**.**

7. Flow rate and velocity relationship

The conversion relationship between flow Q(L/H) and flow velocity V (cm/s) was calculated by formula (S1).

$$v = \frac{0.278Q}{NWt} \quad \text{(S1)}$$

Where, N is the logarithm of the membrane in the membrane stack, W is the width of the diaphragm (cm), and T is the thickness of the diaphragm (cm).

The electrodialysis parameters of this experiment are; N = 1;W = 10 cm; t = 0.92 cm.

8. Charge state of amino acids in adsorption experiments

The charge state of amino acids in the adsorption experiment is shown in Table S1

Table S1. Charge state of amino acids.

**Amino Acids**	**Isoelectric Point (pI)**	**Charged Species**
Glutamate (Glu)	3.22	-
Aspartate (Asp)	2.97	-
Leucine (Leu)	5.98	+
Arginine (Arg)	10.76	+
Isoleucine (Ile)	6.02	+
Phenylalanine (Phe)	5.49	+
Alanine (Ala)	6	+
Lysine (Lys)	9.74	+

Threonine (Thr)	6.16	+
Proline (Pro)	6.3	+
Glycine (Gly)	5.97	+
Serine (Ser)	5.68	+
Tyrosine (Tyr)	5.64	+
Methionine (Met)	5.74	+
Histidine (His)	7.59	+

References

1. Ali, M.B.; Mnif, A.; Hamrouni, B. Modelling of the limiting current density of an electrodialysis process by response surface methodology. Ionics 2018, 24, 617-628, doi:10.1007/s11581-017-2214-7.
